# A novel multimeric sCD19‐streptavidin fusion protein for functional detection and selective expansion of CD19‐targeted CAR‐T cells

**DOI:** 10.1002/cam4.4657

**Published:** 2022-05-27

**Authors:** Hui Lian, Jinhong Jiang, Yao Wang, Xiaoxiao Yu, Rong Zheng, Jing Long, Mengjie Zhou, Shirong Zhou, Cheng Wei, Ai Zhao, Jimin Gao

**Affiliations:** ^1^ The First People's Hospital of Linping District Hangzhou Zhejiang China; ^2^ Zhejiang Provincial Key Laboratory for Technology and Application of Model Organisms, Key Laboratory of Laboratory Medicine, Ministry of Education, School of Laboratory Medicine and Life Sciences Wenzhou Medical University Wenzhou Zhejiang China; ^3^ Department of Hematology, Lishui People's Hospital Sixth Affiliated Hospital of Wenzhou Medical University Lishui Zhejiang China; ^4^ Department of Geriatric, Affiliated Hangzhou First People's Hospital Zhejiang University School of Medicine Hangzhou Zhejiang China; ^5^ Zhejiang Qixin Biotech Wenzhou China

**Keywords:** CAR‐T cells, CD19, fusion protein, streptavidin

## Abstract

**Background:**

CARs are engineered receptors comprising an immunoglobulin single‐chain variable fragment (scFv) that identifies and binds to the target antigen, a transmembrane domain, and an intracellular T‐cell signaling domain. CD19 is a B lineage‐specific transmembrane glycoprotein and is expressed in more than 95% of B‐cell malignancies. Streptavidin (SA) is a homo‐tetrameric protein derived from *Streptomyces avidinii*, which can bind four biotin molecules with an extremely high affinity at a Kd value of 10^‐15^ M.

**Aims:**

The aim of the study is to generate a novel soluble multimeric fusion protein, sCD19‐streptavidin (sCD19‐SA) for functional detection and selective expansion of CD19‐targeted CAR‐T cells.

**Methods:**

The fusion proteins CD19‐SA was expressed in CHO cells and purified by use of Ni‐nitrilotriacetic acid agarose beads.

**Results:**

A novel fusion protein (sCD19‐SA) was generated, consisting of the extracellular domain of human CD19 and the core region of SA, and could be used to functionally detect CD19‐targeted CAR‐T cells. Furthermore, this protein was demonstrated to form multimers to activate CAR‐T cells to induce their selective expansion. Importantly, sCD19‐SA‐stimulated CD19‐targeted CAR‐T cells could improve antitumor effects in vivo.

**Conclusions:**

Our study has highlighted the potential of utilizing antigen‐SA fusion proteins such as sCD19‐SA for CAR‐T therapy for the functional detection of CAR expression and selective expansion of CAR‐T cells.

## Graphical abstract

We generated a novel soluble multimeric fusion protein sCD19‐streptavidin (sCD19‐SA) consisting of the extracellular domain of CD19 and the core streptavidin, which aimed to functionally detect and activate the fourth‐generation CD19‐targeted CAR‐T cells. This work highlights the potential to utilize antigen‐SA fusion proteins such as sCD19‐SA for CAR‐T immunotherapy by functional detection of CAR expression and selective expansion of CAR‐T cells.

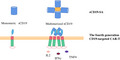

## 1 INTRODUCTION

Recently, cancer immunotherapy has resulted in long‐lasting remission and proved to be an effective therapy in animal studies and clinical trials.^1^, ^2^, ^3^ Chimeric antigen receptor T (CAR‐T) cells have shown remarkable efficacy in treating hematologic malignancies, such as B‐cell malignancies.^4^, ^5^ CARs are engineered receptors comprising an immunoglobulin single‐chain variable fragment (scFv) that identifies and binds to the target antigen, a transmembrane domain, and an intracellular T‐cell signaling domain.^6^ CAR construct design incorporates CD28 and/or 4‐1BB co‐stimulation to enhance therapeutic effects in vivo.^7^, ^8^, ^9^, ^10^, ^11^ Furthermore, fourth‐generation CAR‐T cells engineered to express IL‐7 and CCL19 showed greatly improved antitumor efficacy.^12^ Similarly, we generated CD19‐targeted CAR‐T cells that secrete CCL19 to enhance CCR7^+^ immune cell infiltration.

A previous study reported that soluble cross‐linking antibodies can initiate events similar to proximal TCR signaling in T cells.^13^, ^14^, ^15^ A recent study has shown that soluble multimeric ligands can spontaneously bind up to two or more CARs to trigger CAR signaling.^6^ CD19 is a B lineage‐specific transmembrane glycoprotein of approximately 95 kDa and is expressed in more than 95% of B‐cell malignancies. Stringent lineage restriction has promoted CD19 as an ideal target for CAR‐T therapy.^16^ Streptavidin (SA) is a homo‐tetrameric protein derived from *Streptomyces avidinii*, which can bind four biotin molecules with an extremely high affinity at a Kd value of 10^−15^ M, which is 10^3^–10^6^‐fold higher than the classical antigen–antibody interaction.^17^, ^18^, ^19^, ^20^, ^21^, ^22^, ^23^, ^24^ Owing to its ability to form homotetramers, SA also serves as a multimerization platform to increase the avidity of single‐chain antibodies.^25^, ^26^, ^27^, ^28^

In this study, we generated a novel fusion protein, sCD19‐SA, which consists of the extracellular domain of human CD19 and the core region of SA, for the functional detection and activation of CD19‐targeted CAR‐T cells. Furthermore, we demonstrated that this protein could form multimers to activate CAR‐T cells to induce their selective expansion. Importantly, sCD19‐SA‐stimulated CD19‐targeted CAR‐T cells could improve antitumor effects in vivo.

## 2 MATERIALS & METHODS

### 2.1 Plasmid construction

Human peripheral blood mononuclear cells were collected, and total RNA was extracted using TRIzol (Invitrogen). cDNA that encodes CD19ecto (amino acids 1–291) was obtained by RT‐PCR with the following primer pair: 5′‐GAATTCGCCACCATGCCACTC CTCGCCTCC‐3′ (30 nt), which contained an EcoRI site, and 5′‐CCACCAGAGCCTCCTCCACCCTTCCAGCCACCAGTCCTCAGCAG‐3′ (44 nt). Regions encoding mature SA were amplified by PCR from the genomic DNA of *S. avidinii* (ATCC), with the primer pair 5′‐GGTGGAGGAGGCTCTGGTGGAGGCGGTAGCGGAGGCGGAGGGTCGGCCGAGGCCGGCATC‐3′ (60 nt) and 5′‐GCGGCCGCTTAGTGATGGTGATGGTGATGCTGC TGAACGGCGTCG‐3′ (45 nt), which had a NotI site. These two PCR products were used to perform overlapping PCR, and the resultant PCR product was cut using EcoRI and NotI. Finally, the digested product was cloned into the corresponding sites of the pMH3 vector (Novagen). The structure of the sCD19‐SA fusion protein was based on the CD19 natural signal sequence at its N‐terminus, a single 6‐His tag at its C‐terminal, and a flexible glycine/serine‐rich linker: GGT GGA GGC TCT GGT GGA GGC GGT AGC GGA GGC GGA GGG TCG. The resultant pMH3‐sCD19‐SA plasmid contained an sCD19‐SA insert with an actin promoter, a polyA tail, GC‐rich elements, and a neomycin‐resistant marker.

### 2.2 Cell lines

Jurkat clone E6‐1 (TIB‐152), HEK293T (CRL‐11268) and CHO‐S cells were purchased from ATCC. CHO‐S cells were cultured in Dulbecco's modified Eagle's medium (DMEM)/F12 (Gibco) media supplemented with 10% fetal bovine serum (FBS, Gibco), 20 U/ml penicillin, and 20 mg/ml streptomycin at 37°C and 5% CO_2_. HEK293T cells were cultured in DMEM (Gibco) with 10% HI‐FBS, 1% glutamine, 1% HEPES, and 1% sodium pyruvate. Fourth‐generation CD19‐targeted CAR‐Jurkat cells were generated by lentiviral transduction, and transduction efficiency was detected by flow cytometry. Lentiviral particles were produced in HEK293T cells. Human T cells were derived from PBMCs from healthy adults. CD3/CD28 Dynabeads (Thermo Fisher Scientific) were added to stimulate T cells at a ratio of 1:1 (bead: cell) for 2 days. Then, lentiviruses at a ratio of 40:1 of the multiplicity of infection were transduced into the T cells. The resultant T cells were grown in complete X‐VIVO (04‐744Q, Lonza), supplemented with 5% heat‐inactivated human serum (Sigma), 3% glutamine, 1% HEPES, 1% sodium pyruvate, 1 ng/ml IL‐2, and 5 ng/ml IL‐15 (Peprotech). Dynabeads were removed from the culture medium after 7 days. The transduction efficiency was determined using flow cytometry.

### 2.3 Generation of stable transfectants secreting sCD19‐SA

When CHO‐S cells were 60% confluent in the six‐well plates, transfection was performed in a serum‐free medium with 9 μl Lipofectamine 2000 reagent (Invitrogen Life Technologies) mixed with 2 μg of plasmid DNA (pMH3‐sCD19‐SA). After incubation overnight at 37°C, the cells were washed with phosphate‐buffered saline (PBS) and grown in DMEM/F12 medium. Next, the cells were transferred to a 6‐cm cell culture dish after 48 h, and then, G418 (Invitrogen Life Technologies) was added at 1 mg/ml. The selection process for drug‐resistant cells was continued for 15 days, until single‐cell colonies were formed.

### 2.4 Dot blotting

To identify positive cell clones secreting sCD19‐SA protein, cell culture supernatants were collected for dot blotting. When the cells were confluent in 96‐well plates, 5 μl of culture supernatant was collected from each well and dotted on the nitrocellulose filter membrane. After drying, the membrane was placed in the prepared 5% milk blocking solution at 37°C for 1 h and subsequently incubated with anti‐mouse CD19 antibody (Santa Cruz Biotechnology; 1: 2500 dilution in blocking buffer) and horseradish peroxidase‐conjugated anti‐mouse IgG (1: 5000) for 1 h at 37°C. Finally, a 3,3′‐diaminobenzidine (DAB) reaction was performed using a DAB display liquid kit (Boshide Corporation) after extensive washing.

### 2.5 Production and purification of the sCD19‐SA protein

The stable transfectants secreting sCD19‐SA were cultured in DMEM/F12 medium for 72 h, and the supernatant was collected by centrifugation. The sCD19‐SA fusion protein was captured by passing the CHO‐S supernatant through an Ni‐NTA column (Qiagen) according to the manufacturer's instructions. The column was equilibrated with 200 ml of buffer containing 5 mM imidazole, and the sCD19‐SA fusion protein was eluted with 100 mM imidazole. The collected eluate was concentrated and washed with PBS by ultrafiltration (Amicon Ultra‐15 centrifugal units, 50 kDa).

### 2.6 Western blotting

To detect sCD19‐SA, reduced or unreduced samples were loaded and electrophoresed on an 8% sodium dodecyl sulfate‐polyacrylamide gel and subsequently electrotransferred onto a polyvinylidene difluoride membrane (Bio‐Rad). The blotted membrane was blocked with 5% skim milk in Tris‐buffered saline with 0.5% Tween‐20 (TBST) at 37°C for 1 h and subsequently incubated with anti‐mouse CD19 antibody (Santa Cruz Biotechnology) at 1: 2500 dilution in blocking buffer at 37°C for 1 h. The membrane was washed three times with TBST and then incubated in anti‐mouse IgG‐conjugated horseradish peroxidase at 1:5000 for another hour at 37°C. After extensive washing, the DAB reaction was visualized using a DAB display liquid kit (Boshide). Primary antibodies for CD3ζ (BD Biosciences) and anti‐rabbit HRP‐conjugated IgG (Santa Cruz Biotechnology) were used to detect anti‐CD19 CAR.

### 2.7 FITC labeling

The protein to be cross‐linked (concentration ≥1 mg/ml) was dialyzed against sodium carbonate cross‐linking reaction solution three times at 4°C to pH 9.0, and FITC was dissolved in DMSO at a concentration of 1 mg/ml. FITC was slowly added to the antibody solution at a protein: FITC (P:F) ratio of 1 mg:50 μg and reacted in the dark at 4°C for 8 h. After adding 5 mol/L NH_4_Cl to a final concentration of 50 mmol/L, the reaction was terminated at 4°C for 2 h. Free FITC was removed using a G50 column, and the labeled product was identified using a UV spectrophotometer (DU730, HITACHI).

### 2.8 Lentiviral vectors for gene transfection into T cells

Anti‐CD19 scFv was derived from a murine antibody (FMC63) and fused with the transmembrane domain of CD8, cytoplasmic regions of 4‐1BB (CD137), and CD3ζ endodomains to construct a second‐generation CAR. To express human CCL19 concomitantly with CAR, the foot‐and‐mouth‐disease virus 2A peptide sequence was intercalated among genes to construct a fourth‐generation CAR. These CARs were cloned into the pLenti‐vector to obtain recombinant plasmids. HEK‐293 T cells were transfected with pLenti‐CAR and the lentiviral packing plasmids pLP1, pLP2, and pMD2.G by polyethylenimine transfection to obtain lentiviral particles. All sequences were synthesized by Thermo Fisher Scientific.

### 2.9 Flow cytometry and antibody staining

Flow cytometry was performed using a FACSAria II cell counter (BD Biosciences). T cells were assessed for the surface presentation of epitopes with fluorescently labeled monoclonal antibodies for CD69 (BioLegend). In parallel, anti‐CD19 CAR expression was measured using Flow cytometry. First, anti‐CD19 CAR expression was measured with commercial bio‐sCD19‐Fc (ACROBiosystems), followed by staining with secondary APC‐conjugated SA antibody (BioLegend). Anti‐CD19 CAR expression was measured with sCD19‐SA, followed by a secondary PE‐conjugated anti‐SA antibody (BioLegend), and anti‐CD19 CAR expression was directly measured with FITC‐sCD19‐SA. To assess cytokines produced by CAR‐T cells, cells were incubated in 96‐well U‐bottom plates at 10^5^ cells/100 μl media/well for 24 h in the presence of 60 μg/ml sCD19‐SA with 5 μg/ml Brefeldin A (BioLegend), and the hGM‐CSF‐SA fusion protein (prepared in our laboratory) was added to the medium alone as a negative control. Before permeabilization with ice‐cold methanol, the cells were fixed in 1.5% formaldehyde and then incubated with antibodies against FITC‐TNF‐α, PE‐IFN‐γ, and APC‐IL‐2 (all from BioLegend) for flow cytometric analysis. A total of 10^4^ cells were counted by flow cytometry for each experiment.

### 2.10 Measurement of cytokine secretion using enzyme‐linked immunosorbent assay

For detection of cytokine release, CAR‐T cells were incubated in 96‐well U‐bottom plates at 10^5^ cells/100 μl media/well for 24 h in the presence of 60 μg/ml sCD19‐SA, and secretion of cytokines such as IL‐2, TNF‐α, and IFN‐γ in the supernatant was analyzed using an ELISA kit (LIANKE), according to the manufacturer's instructions.

### 2.11 In vitro migration assay

Cell chemotaxis was measured by migration assay using a 3‐μm pore size polycarbonate filter in a 24‐well ultrafiltration chamber (Corning). Supernatants (600 μl) of second‐generation anti‐CD19 CAR‐T cells, fourth‐generation anti‐CD19‐CCL19 CAR‐T cells, and untransduced T cells were added to the lower chamber as a control. All the above cells were co‐cultured with 60 μg/ml sCD19‐SA for 24 h. CFSE‐labeled responder T cells (2 × 10^5^) were placed in the upper chamber and incubated at 37°C for 3 h. The number of CFSE‐labeled responder T cells migrating from the upper chamber to the lower chamber was assessed using a fluorescence microscope (Olympus).

### 2.12 T‐cell expansion assay

T cells (10^5^/100 μl media/well) were cultured with or without 60 μg/ml sCD19‐SA, and the media was replaced every 2–3 days. T‐cell suspensions were mixed with 0.4% trypan blue, and 20 μl was taken to perform a viable cell count. Viable cell density was estimated using a Count Star (Inno‐Alliance Biotech).

### 2.13 sCD19‐SA‐induced T‐cell expansion

Anti‐CD19‐CCL19‐GFP CAR‐T cells (10^5^/100 μl media/well) were stimulated in the presence or absence of 60 μg/ml sCD19‐SA for 7 days. To monitor the specific expansion of fourth‐generation CD19‐targeted CAR‐T cells, cells were collected for flow cytometric analysis on day 3 or 7.

### 2.14 In vivo analysis of CAR‐T activity response to sCD19‐SA

NSG mice (5–6 weeks of age) were purchased from GemPharmatech Company and injected intravenously with 4 × 10^6^ Raji cells stably expressing luciferase‐GFP (Raji‐luc‐GFP). Then, 4 × 10^6^ CAR‐T cells cultured with or without sCD19‐SA and mock‐T cells as a control were injected intravenously on day 7. Tumor burden was measured using the IVIS Spectrum BL through intraperitoneal injection of 150 mg/kg d‐luciferin. Imaging was performed on days 3, 10, 17, and 24 to monitor tumor progression. All reagents and instruments were obtained from PerkinElmer.

### 2.15 Statistical analysis

The experiments were independently conducted three or more times. Student's t‐test was used to compare continuous variables between the two groups and analyzed using GraphPad Software 6.0. To compare repeated measures of means with respect to a single variable, one‐way repeated‐measures analysis of variance was used. Results were presented as the mean ± standard error of the mean, and mean values were expressed with horizontal bars. Statistical significance was set at *p* < 0.05.

## 3 RESULTS

### 3.1 Expression and purification of the sCD19‐SA fusion protein

**FIGURE 1:**
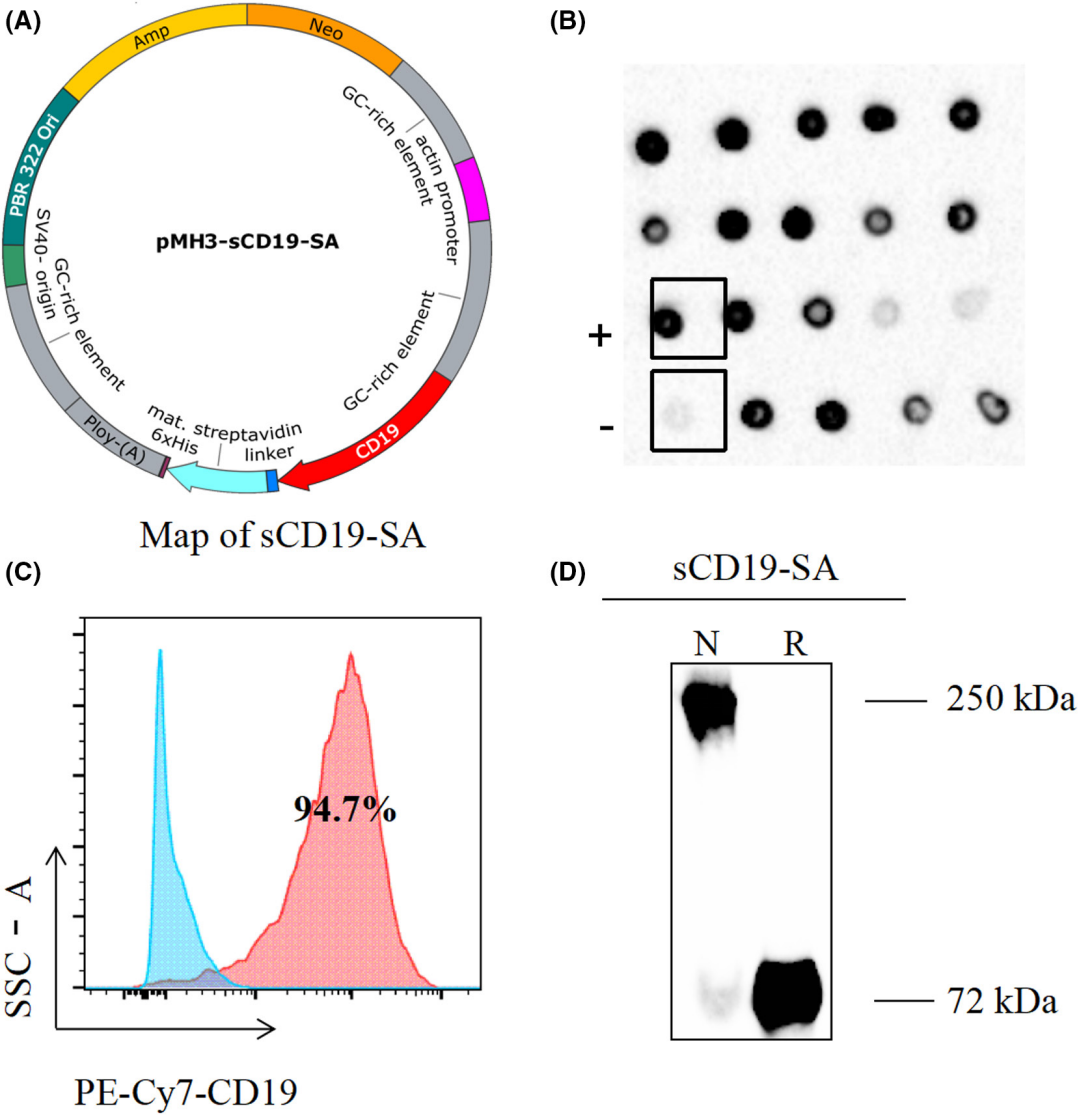
Generation of the sCD19‐SA fusion protein. (A) Map of the constructed plasmid pMH3‐sCD19‐SA. (B) Expression and identification of the sCD19‐SA fusion protein. The vector encoding sCD19‐SA was transfected into CHO cells. The supernatant was analyzed using a Dot Blot. The supernatant of negative CHO cells was used as a negative control (−) and commercial biotin‐sCD19‐FC as the positive control (+). (C) Intracellular staining of cells transfected with a vector encoding sCD19‐SA CHO cells treated with a transport inhibitor using anti‐PE‐Cy7 CD19 for flow cytometric analysis. (D) CHO supernatants were detected by western blot in non‐reduced (lane 1) or reduced (lane 2) conditions, using anti‐human CD19 antibody. Size markers (in kDa) are shown on the left

To obtain sCD19‐SA, the cDNA encoding the extracellular region of CD19 was fused with that of the core SA with a 24 amino acid extension at the C‐terminus to form a multimer, which may improve its hydrophilicity (Figure 1A). The fusion protein of sCD19‐SA was encoded by 459 amino acid residues, including 19 residues of the CD19 signal peptide, 272 residues of the CD19 extracellular region, 147 residues of SA, and a 15‐residue linker between the extracellular region of CD19 and core SA with a 24 amino acid extension at the C‐terminus containing a single 6‐His tag. Thus, the mature secretory sCD19‐SA monomer has 440 amino acid residues, with a molecular weight of 49440.87 Da.

To express sCD19‐SA, the pMH3 plasmid was transfected into CHO‐S cells, and the stable clones were selected by G418. Efficient transfectants expressing a high level of the sCD19‐SA fusion protein were screened by dot blotting and intracellular specific staining (Figure 1B,C). The product size was determined by western blotting, and specific protein bands of approximately 250 and 72 kDa were detected by the purification of CHO supernatant under non‐reducing and reducing conditions, respectively, confirming that the sCD19‐SA fusion protein was expressed as a multimer (Figure 1D). Compared to the molecular weight of 49 kDa inferred from the amino acid sequence in the reduced form, the apparent molecular weight of 72 kDa may result from post‐translational modifications such as glycosylation. As expected, the sCD19‐SA fusion protein was found to be multimerized in a non‐reducing form.

### 3.2 Generation of CD19‐targeted CAR‐T cells secreting CCL19

**FIGURE 2:**
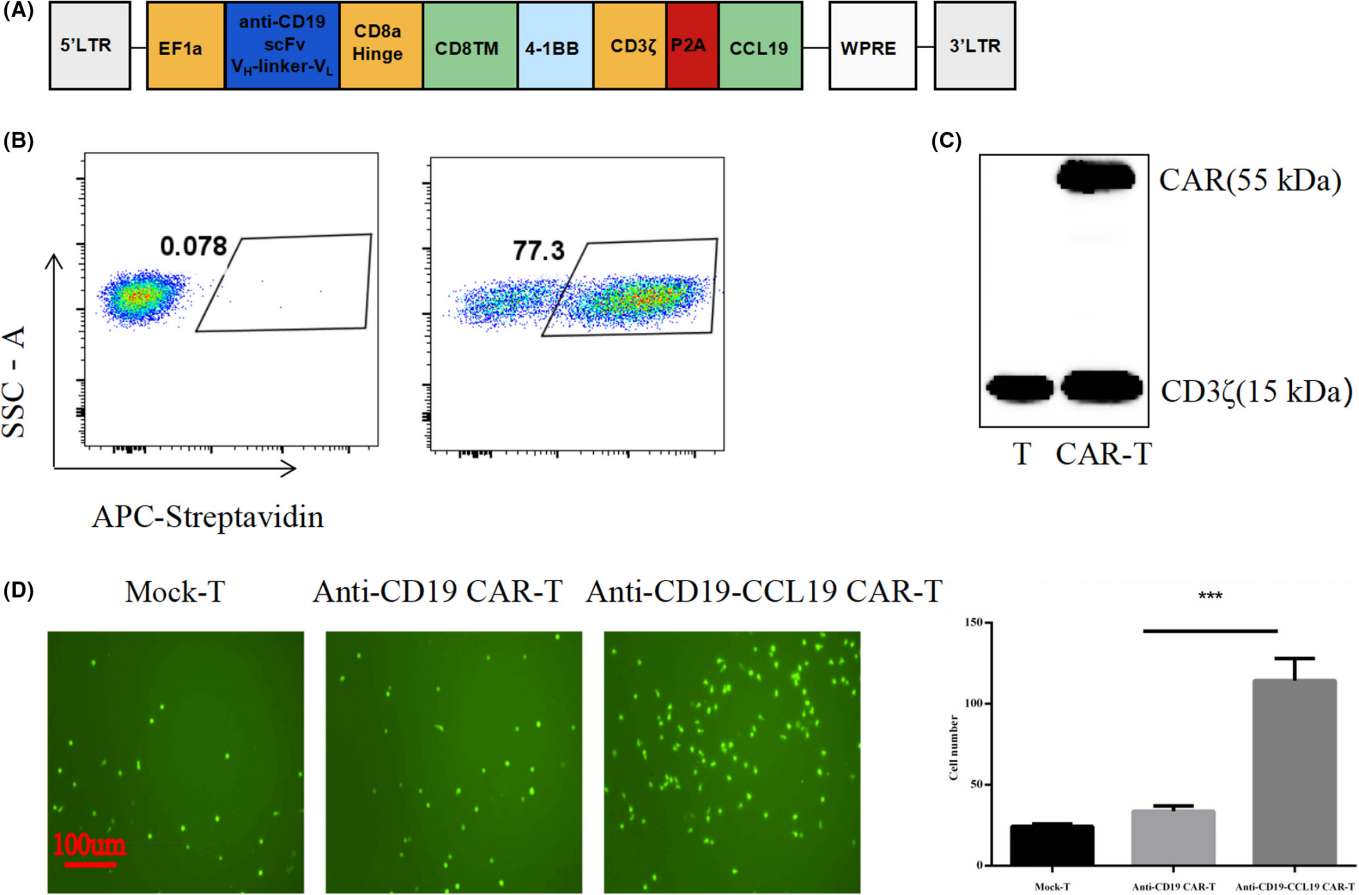
Generation of the fourth‐generation CD19‐targeted CAR‐T cells. (A) The fourth‐generation anti‐CD19 CAR comprised an extracellular CD19‐binding scFv linked to the CD8 hinge and transmembrane 4‐1BB and CD3ζ endodomains. CCL19 was separated from P2A cells. P2A, ‘self‐cleaving’ peptide; TM, transmembrane domain; VH, heavy‐chain variable domain; VL, light chain variable domain. (B) Expression rate of CARs at MOI = 40. Anti‐CD19 CAR was detected by flow cytometry using commercial biotin‐sCD19‐FC, followed by staining with a secondary APC‐conjugated streptavidin antibody. (C) CAR expression in T lymphocytes was validated by western blot analysis after transduction. Lysates of untransduced T cells (lane 1), anti‐CD19‐CCL19 CAR transduced (lane 2). (D) Transwell assays were conducted to detect the migration capacity of T cells transfected with anti‐CD19 CAR, anti‐CD19‐CCL19 CAR, with untransduced T cells (Mock‐T) used as a control, and the cells were cultured with 60 μg/ml sCD19‐SA for 24 h. The number of CFSE‐labeled responder T cells migrating from the upper chamber to the lower chamber in different supernatants was analyzed using a fluorescence microscope. Data points from *n* = 3 biologically independent cell cultures are presented as mean ± 1 deviation, ***p < 0.001

Recently, second‐generation CAR‐T cells with an intracellular signaling domain composed of CD3ζ and CD28 or 4‐1BB costimulatory molecules have been used in many clinical trials, and fourth‐generation CAR‐T technology is currently under development and has been involved in the integrated expression of immune regulators.^1^ CCL19 is a chemokine for CCR7^+^ T cells and DCs.^29^, ^30^, ^31^ Fourth‐generation CD19‐targeted CAR‐T cells were engineered to express CCL19 to improve chemotactic activity (Figure 2A). Flow cytometry and western blotting illustrated the successful construction of the fourth‐generation anti‐CD19 CAR (Figure 2B,C). When the MOI was 40, the expression of CAR reached 80% in human T cells. Transwell migration analysis showed that there were more responder T cells incubated with the fourth‐generation CD19‐targeted CAR‐T cells than those incubated with the second‐generation anti‐CD19 CAR‐T cells, which is consistent with the findings of previous reports^12^ (Figure 2D), and that sCD19‐SA fusion protein did not affect CCL19 function of the fourth‐generation CD19‐targeted CAR‐T cells.

### 3.3 Functional detection of anti‐CD19 CAR by sCD19‐SA

**FIGURE 3:**
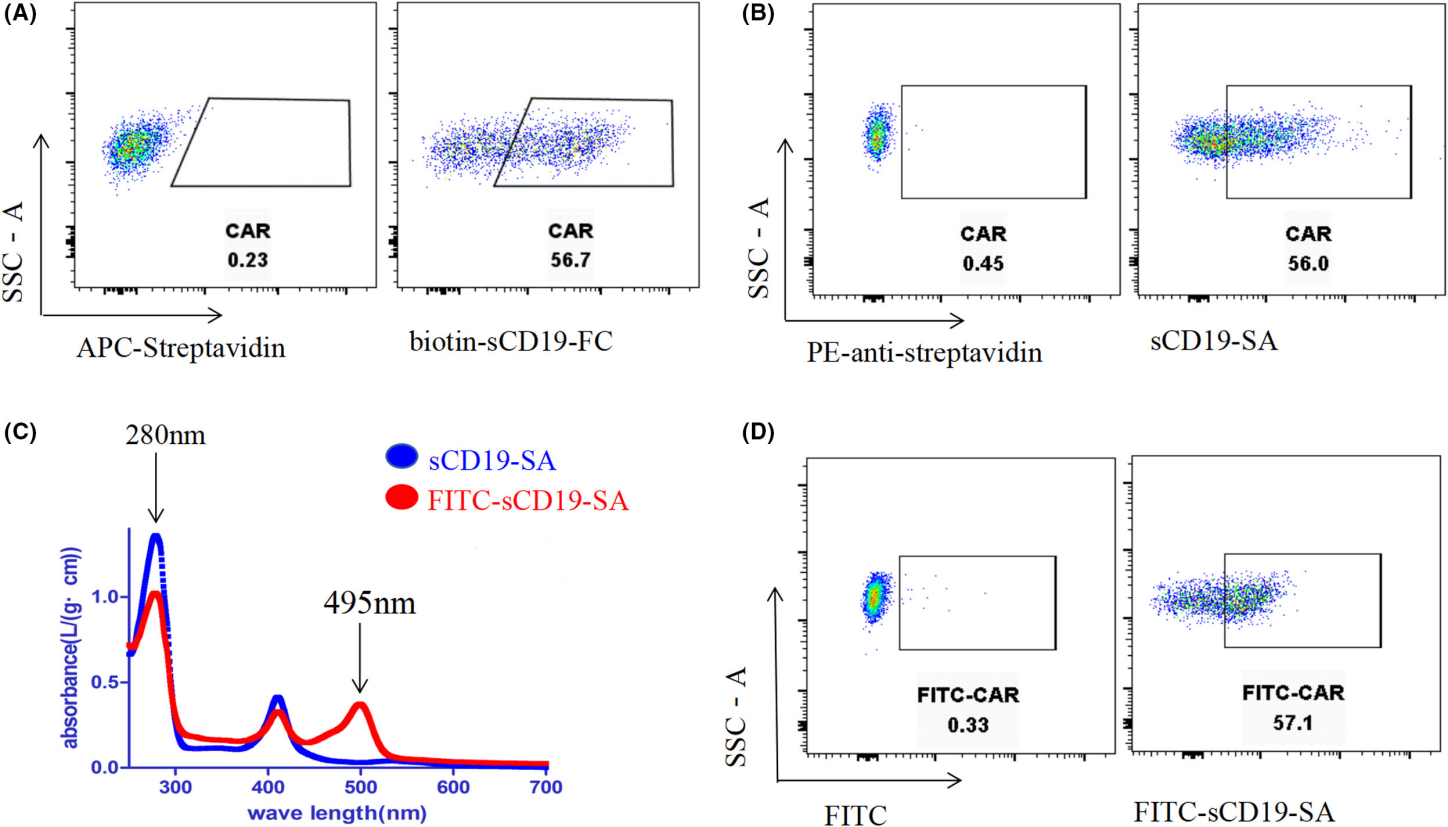
Detection of anti‐CD19 CAR by sCD19‐SA. (A) Anti‐CD19 CAR expression was detected using flow cytometry with commercial biotin‐sCD19‐FC. Fourth‐generation CD19‐targeted CAR‐Jurkat (Anti‐CD19‐CCL19 CAR‐Jurkat) cells were stained with commercial biotin‐sCD19‐FC, followed by staining with a secondary APC‐conjugated streptavidin antibody. (B) Fourth‐generation CD19‐targeted CAR‐Jurkat cells were stained with sCD19‐SA, followed by a secondary PE‐conjugated anti‐streptavidin antibody. (C) The sCD19‐SA protein was labeled with FITC. (D) Fourth‐generation CD19‐targeted CAR‐Jurkat cells were stained with FITC‐sCD19‐SA without secondary antibodies

To confirm whether the sCD19‐SA fusion protein exhibited native binding activity to the anti‐CD19 scFv derivative in the CD19‐targeted CAR‐T cells, flow cytometry was performed and the results showed that the functional detection of CAR‐expressing Jurkat T cells with sCD19‐SA was equivalent to that with a commercial product bio‐sCD19‐Fc, which occurs in the presence of a secondary conjugated antibody (Figure 3A,B). To simplify the detection of CAR expression, we labeled sCD19‐SA with FITC and purified FITC‐sCD19‐SA using a G25 column, which was found to have an absorption peak at 280 nm, with a FITC absorption peak at 495 nm (Figure 3C). FITC‐sCD19‐SA was demonstrated to directly detect the expression of anti‐CD19 CAR without a secondary conjugated antibody, which was similar to the result of the indirect detection described above (Figure 3D). In conclusion, sCD19‐SA specifically detected the functional expression of anti‐CD19 CAR.

### 3.4 Activation of CD19‐targeted CAR‐T cells by sCD19‐SA

**FIGURE 4:**
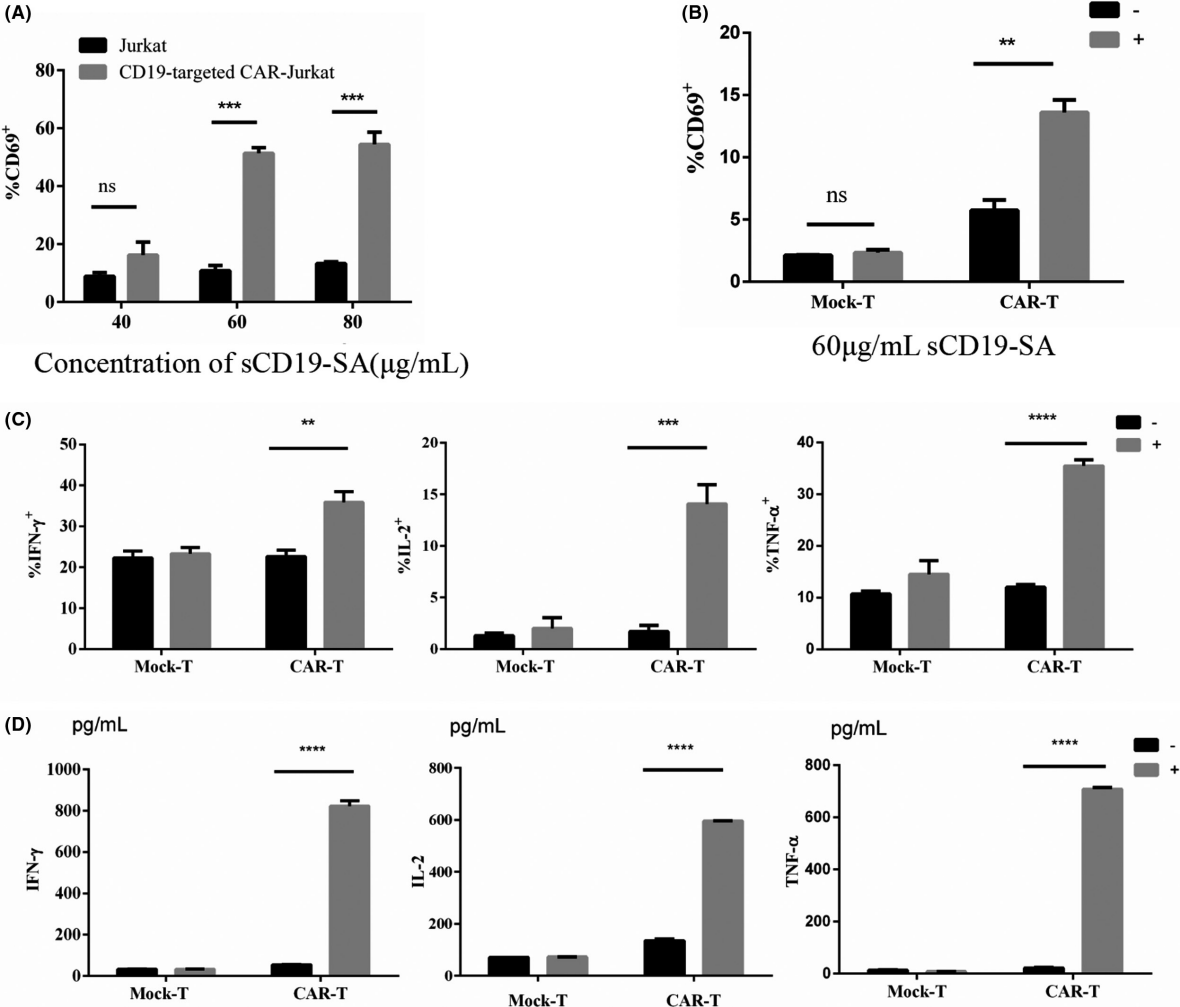
Activation of fourth‐generation CD19‐targeted CAR‐T cells by sCD19‐SA. (A) Fourth‐generation CD19‐targeted CAR‐Jurkat and Jurkat cells were incubated for 24 h with different concentrations of sCD19‐SA (40–80 μg/ml). The percentage of CD69‐expressing cells was detected using flow cytometry with PE‐Cy7‐conjugated anti‐CD69 antibody. (B) Primary human T cells (Mock‐T) and anti‐CD19‐CCL19 CAR‐T cells were incubated for 24 h with 60 μg/ml sCD19‐SA (+), and the hGM‐CSF‐SA fusion protein was added to the media alone as a negative control (−). (C) TNF‐α, IL‐2, and IFN‐γ were assessed by intracellular cytokine staining of T cells or anti‐CD19‐CCL19 CAR‐T cells stimulated by sCD19‐SA at 60 μg/ml total protein (+). (D) ELISA quantified anti‐CD19‐CCL19 CAR‐T cells secretion of cytokine in response to sCD19‐SA for 24 h. In (A–D), data points from *n* = 3 biologically independent cell cultures are presented as means ± 1 SD, **p < 0.01, ***p < 0.001, ****p < 0.0001

Since the multimeric antigen sCD19‐SA fusion protein could specifically bind to CD19‐targeted CAR‐T cells to detect CAR expression, we further explored whether this protein could induce CAR activation. CD69 is a human transmembrane C‐type lectin protein expressed as an early activation marker on the surface of hematopoietic stem cells, T cells, and many other immune cells.^32^, ^33^ To determine whether the sCD19‐SA fusion could selectively activate the anti‐CD19 CAR‐expressing Jurkat cells used as an antigen, the fusion protein was tested at three different concentrations (40, 60, and 80 μg/ml). The concentration of sCD19‐SA up to 60 μg/ml was shown to trigger CD69 upregulation in Jurkat T cells expressing anti‐CD19 CAR (Figure 4A). Similarly, CD19‐targeted CAR‐T cells upregulated the expression of CD69 in response to sCD19‐SA (Figure 4B). Furthermore, the sCD19‐SA fusion protein could stimulate CD19‐targeted CAR‐T cells to secrete immunostimulatory cytokines, such as IFN‐γ, IL‐2, and TNF‐α (Figure 4C,D).

### 3.5 Selective expansion of CD19‐targeted CAR‐T cells by sCD19‐SA

**FIGURE 5:**
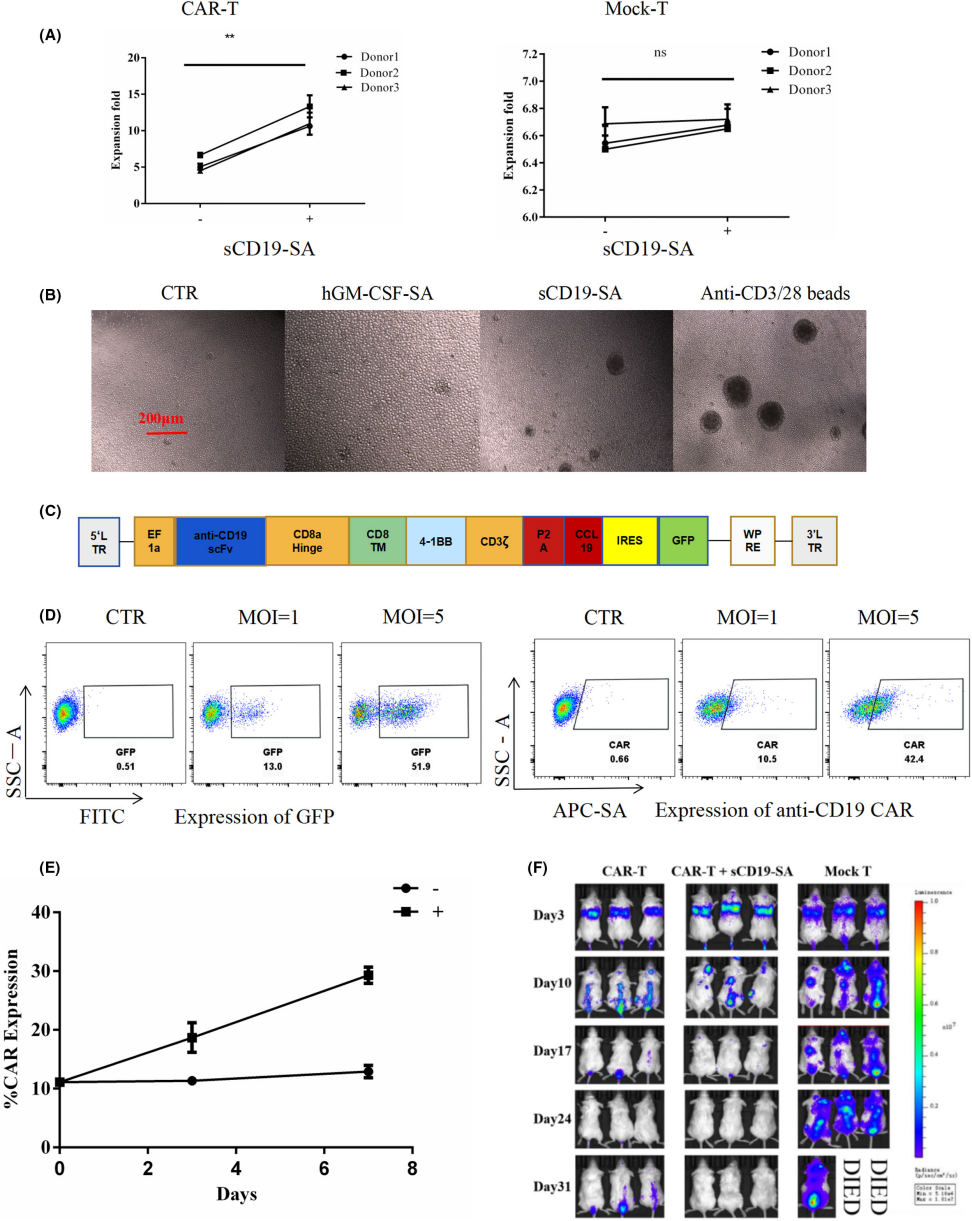
Activation‐induced specific expansion of fourth‐generation CD19‐targeted CAR‐T cells by sCD19‐SA. (A) Expansion folds of anti‐CD19‐CCL19 CAR‐T cells (Left) and primary human T cells (Right) in the presence (+) or absence of sCD19‐SA, the hGM‐CSF‐SA fusion protein was added in the media alone as negative control (−). (B) The cell density of anti‐CD19‐CCL19 CAR‐T cells responds to media alone, hGM‐CSF‐SA, sCD19‐SA and anti‐CD3/28 beads (200x). (C) The fourth‐generation anti‐CD19 CAR contains a GFP tag was separated with IRES. (D) Four days after lentiviral transduction at different rates (MOI = 1 and MOI = 5), GFP and anti‐CD19 CAR expression on anti‐CD19‐CCL19‐GFP CAR‐T cells were detected using flow cytometry. Anti‐CD19 CAR was measured by flow cytometry with commercial biotin‐sCD19‐FC fusion protein. Cells were stained with biotin‐sCD19‐FC, and stained with a secondary APC‐conjugated streptavidin antibody. The measurement of CAR expression by GFP was shown to be repeatable. (E) Anti‐CD19‐CCL19‐GFP CAR‐T cells with the initial GFP expression of 11.1% were stimulated with (+) or without (−) sCD19‐SA for 7 days. GFP expression after stimulation was measured by flow cytometry on days 3 and 7. (F) NSG mice were injected intravenously with 4 × 10^6^ Raji‐Luc‐GFP cells on day 0 and received 4 × 10^6^ CAR‐T cells cultured with sCD19‐SA, CAR‐T cells, and Mock‐T cells as a control on day 7. Imaging analysis to monitor tumor progression was performed on days 3, 10, 17, and 24

Considering the proliferative capacity of CAR‐T cells is critical for cancer immunotherapy, we examined whether the sCD19‐SA fusion protein promoted the expansion of CD19‐targeted CAR‐T cells. In our study, the sCD19‐SA fusion protein significantly enhanced the activation and proliferation of fourth‐generation CD19‐targeted CAR‐T cells (Figure 5A,B). To further verify whether sCD19‐SA could stimulate the specific clonal expansion of CD19‐targeted CAR‐T cells in a mixture of anti‐CD19 CAR‐transduced and ‐untransduced cells, we constructed an anti‐CD19 CAR with a GFP tag to demonstrate the expression of CAR by flow cytometry (Figure 5C). CAR expression could be reliably measured through GFP intensity, regardless of variance in lentiviral transduction efficiency (Figure 5D). Indeed, sCD19‐SA increased the proportion of anti‐CD19‐CCL19‐GFP CAR‐T cells from 11.1% to 30.1% on day 7 by selectively promoting the proliferation of CD19‐targeted CAR‐T cells (Figure 5E). To determine the anti‐tumor activity of sCD19‐SA‐stimulated CD19‐targeted CAR‐T cells in vivo, NSG mice were inoculated with Raji‐Luc‐GFP cells as a xenograft model of Hodgkin's lymphoma. Then, 4 × 10^6^ CAR‐T cells cultured with sCD19‐SA, CAR‐T cells, and mock‐T cells were injected intravenously on day 7. We found that mice treated with sCD19‐SA‐stimulated CD19‐targeted CAR‐T cells exhibited better tumor regression than CAR‐T cells without stimulated by sCD19‐SA (Figure 5F).

## 4 DISCUSSION

We generated a soluble multimeric sCD19‐SA fusion protein. sCD19‐SA is a novel multimer resulting from SA, with an ability to form a homotetramer. The novel multimeric fusion protein sCD19‐SA, as a prototype of the antigen‐SA platform, can be used for the functional detection of anti‐CD19 CAR and further selective activation and expansion of CD19‐targeted CAR‐T cells to improve their antitumor effects in vivo.

Previous studies have provided no evidence to indicate that CAR signaling can be triggered by the soluble form of target antigens, such as CD30, mesothelin, carcinoembryonic antigen, and Lewis Y antigen.^34^, ^35^, ^36^, ^37^, ^38^ However, recent studies have shown that soluble ligands in dimers or multimers can simultaneously bind two or more CARs to trigger CAR signaling. The CAR structure is very important because the binding affinity for its ligand should be strong enough to trigger CAR signaling and the CAR with its soluble ligand must have sufficient mechanical rigidity to transfer the tension to the intracellular signaling domain.^6^ As shown above, the multimeric sCD19‐SA fusion protein exhibited a strong capacity to activate anti‐CD19 CAR. In addition, our study indicated that the appropriate combination of CAR with some immune regulators such as CCL19 could endow CAR‐T cells with novel functions such as chemotactic activity for CCR^+^ T cells and DCs.

It is essential to detect CAR expression for CAR‐T therapy, and there are several assays to detect different structural regions of CAR, with those targeting CAR antigen‐binding sites such as anti‐CD19 scFv, anti‐Fab, or anti‐hinge regions. The detection of antigen‐binding sites for CAR expression is the best method to achieve this. Accordingly, the sCD19‐SA fusion protein, a multimeric antigen, was found to successfully detect the expression of CAR in CD19‐targeted CAR‐T cells by flow cytometry. Furthermore, without a secondary antibody, the expression of CAR could be detected more quickly and easily using the SA‐antigen fusion protein labeled with FITC.

After proving that the sCD19‐SA fusion protein could specifically bind to anti‐CD19CAR, we further investigated whether the fusion protein could induce CAR activation. Our data showed that sCD19‐SA could activate CD19‐targeted CAR‐T cells as a multimer and promote cytokine secretion. The mechanism behind the activation was found to be multimeric binding‐induced CAR conformational changes, resulting in a sufficiently strong mechanical force transfer to the intracellular signaling domain of CAR to cause CAR activation.^6^ We also observed that activated T cells could be reactivated to proliferate by the related antigen.^38^ Similarly, our study confirmed that the multimeric fusion protein sCD19‐SA could induce selective expansion of CD19‐targeted CAR‐T cells in vitro. It is well‐known that obtaining sufficient CAR‐T cells is critical for successful immunotherapy, but CAR‐T cells are stimulated to achieve moderate expansion with anti‐CD3/anti‐CD28 antibodies.^39^ Here, we demonstrated that CD19‐targeted CAR‐T cells could be activated by sCD19‐SA, consistent with the findings of previous reports,^38^, ^40^, ^41^ and were thus induced to selectively expand in vitro. Importantly, sCD19‐SA‐stimulated CD19‐targeted CAR‐T cells could improve the antitumor effect in vivo.

Taken together, our work highlights the potential of utilizing antigen‐SA fusion proteins such as sCD19‐SA in CAR‐T therapy as a method for functional detection of CAR expression and selective expansion of CAR‐T cells.

## CONFLICT OF INTEREST

No conflict of interest, financial or otherwise is declared.

## AUTHOR CONTRIBUTIONS

Hui Lian, Cheng Wei, Ai Zhao, and Jimin Gao concieved and designed the project. Hui Lian, Jinhong Jiang, Yao Wang, Xiaoxiao Yu, Rong Zheng, Jing Long, Mengjie Zhou, and Shirong Zhou performed the experiments and acquired data. Hui Lian, Xiaoxiao Yu, and Yao Wang were involved in data analysis and interpretation. Drs. Ai Zhao and Jinhong Jiang have been carrying out the clinical trials of CAR‐T therapy by use of the protocol derived from the study. Hui Lian and Jinhong Jiang wrote the manuscript. Ai Zhao and Jimin Gao revised the manuscript. All authors approved the final version of the manuscript.

## COMPLIANCE WITH ETHICAL STANDARDS

The present study was approved by the Ethical Committee of Wenzhou Medical University in Wenzhou, China. The peripheral blood samples used were obtained from healthy donors who had provided their informed consent. All procedures were in accordance with the Declaration of Helsinki.

## Data Availability

The raw data supporting the conclusions of this article will be made available by the authors, without undue reservation.

## References

[1] Perez‐Amill L , Marzal B , Urbano‐Ispizua A , Juan M , Martin‐Antonio B . CAR‐T cell therapy: a door is open to find innumerable possibilities of treatments for cancer patients. Turk J Haematol. 2018;35(4):217‐228. doi:10.4274/tjh.2018.0196 30185400 PMC6256819

[2] Si W , Li C , Wei P . Synthetic immunology: T‐cell engineering and adoptive immunotherapy. Synth Syst Biotechnol. 2018;3(3):179‐185. doi:10.1016/j.synbio.2018.08.001 30345403 PMC6190530

[3] Zhang T , Cao L , Xie J , et al. Efficiency of CD19 chimeric antigen receptor‐modified T cells for treatment of B cell malignancies in phase I clinical trials: a meta‐analysis. Oncotarget. 2015;6(32):33961‐33971. doi:10.18632/oncotarget.5582 26376680 PMC4741817

[4] Srivastava S , Riddell SR . Engineering CAR‐T cells: design concepts. Trends Immunol. 2015;36(8):494‐502. doi:10.1016/j.it.2015.06.004 26169254 PMC4746114

[5] Duong CP , Yong CS , Kershaw MH , Slaney CY , Darcy PK . Cancer immunotherapy utilizing gene‐modified T cells: from the bench to the clinic. Mol Immunol. 2015;67(2 Pt A):46‐57. doi:10.1016/j.molimm.2014.12.009 25595028

[6] Chang ZL , Lorenzini MH , Chen X , Tran U , Bangayan NJ , Chen YY . Rewiring T‐cell responses to soluble factors with chimeric antigen receptors. Nat Chem Biol. 2018;14(3):317‐324. doi:10.1038/nchembio.2565 29377003 PMC6035732

[7] Knochelmann HM , Smith AS , Dwyer CJ , Wyatt MM , Mehrotra S , Paulos CM . CAR T cells in solid tumors: blueprints for building effective therapies. Front Immunol. 2018;9:1740. doi:10.3389/fimmu.2018.01740 30140266 PMC6094980

[8] Henke KG , Badr MS , Skatrud JB , Dempsey JA . Load compensation and respiratory muscle function during sleep. J Appl Physiol. 1992;72(4):1221‐1234. doi:10.1152/jappl.1992.72.4.1221 1592708

[9] Nelson EE , Guyer AE . The development of the ventral prefrontal cortex and social flexibility. Dev Cogn Neurosci. 2011;1(3):233‐245. doi:10.1016/j.dcn.2011.01.002 21804907 PMC3143481

[10] Brentjens RJ , Davila ML , Riviere I , et al. CD19‐targeted T cells rapidly induce molecular remissions in adults with chemotherapy‐refractory acute lymphoblastic leukemia. Sci Transl Med. 2013;5(177):177ra38. doi:10.1126/scitranslmed.3005930 PMC3742551 23515080

[11] Grupp SA , Kalos M , Barrett D , et al. Chimeric antigen receptor‐modified T cells for acute lymphoid leukemia. N Engl J Med. 2013;368(16):1509‐1518. doi:10.1056/NEJMoa1215134 23527958 PMC4058440

[12] Adachi K , Kano Y , Nagai T , Okuyama N , Sakoda Y , Tamada K . IL‐7 and CCL19 expression in CAR‐T cells improves immune cell infiltration and CAR‐T cell survival in the tumor. Nat Biotechnol. 2018;36(4):346‐351. doi:10.1038/nbt.4086 29505028

[13] Letourneur F , Klausner RD . T‐cell and basophil activation through the cytoplasmic tail of T‐cell‐receptor zeta family proteins. Proc Natl Acad Sci USA. 1991;88(20):8905‐8909. 1833767 10.1073/pnas.88.20.8905 PMC52619

[14] Irving BA , Weiss A . The cytoplasmic domain of the T cell receptor zeta chain is sufficient to couple to receptor‐associated signal transduction pathways. Cell. 1991;64(5):891‐901. 1705867 10.1016/0092-8674(91)90314-o

[15] Romeo C , Seed B . Cellular immunity to HIV activated by CD4 fused to T cell or fc receptor polypeptides. Cell. 1991;64(5):1037‐1046. 1900456 10.1016/0092-8674(91)90327-u

[16] Makita S , Yoshimura K , Tobinai K . Clinical development of anti‐CD19 chimeric antigen receptor T‐cell therapy for B‐cell non‐Hodgkin lymphoma. Cancer Sci. 2017;108(6):1109‐1118. doi:10.1111/cas.13239 28301076 PMC5480083

[17] Lim KH , Huang H , Pralle A , Park S . Engineered streptavidin monomer and dimer with improved stability and function. Biochemistry. 2011;50(40):8682‐8691. doi:10.1021/bi2010366 21892837

[18] Wu SC , Wong SL . Intracellular production of a soluble and functional monomeric streptavidin in Escherichia coli and its application for affinity purification of biotinylated proteins. Protein Expr Purif. 2006;46(2):268‐273. doi:10.1016/j.pep.2005.10.006 16289701

[19] Wu SC , Wong SL . Engineering soluble monomeric streptavidin with reversible biotin binding capability. J Biol Chem. 2005;280(24):23225‐23231. doi:10.1074/jbc.M501733200 15840576

[20] Sano T , Pandori MW , Chen X , Smith CL , Cantor CR . Recombinant core streptavidins. A minimum‐sized core streptavidin has enhanced structural stability and higher accessibility to biotinylated macromolecules. J Biol Chem. 1995;270(47):28204‐28209. 7499314 10.1074/jbc.270.47.28204

[21] Dundas CM , Demonte D , Park S . Streptavidin‐biotin technology: improvements and innovations in chemical and biological applications. Appl Microbiol Biotechnol. 2013;97(21):9343‐9353. doi:10.1007/s00253-013-5232-z 24057405

[22] Lim KH , Huang H , Pralle A , Park S . Stable, high‐affinity streptavidin monomer for protein labeling and monovalent biotin detection. Biotechnol Bioeng. 2013;110(1):57‐67. doi:10.1002/bit.24605 22806584

[23] O'Sullivan VJ , Barrette‐Ng I , Hommema E , et al. Development of a tetrameric streptavidin mutein with reversible biotin binding capability: engineering a mobile loop as an exit door for biotin. PLoS One. 2012;7(4):e35203. doi:10.1371/journal.pone.0035203 22536357 PMC3334968

[24] Wu SC , Hassan Qureshi M , Wong SL . Secretory production and purification of functional full‐length streptavidin from *Bacillus subtilis* . Protein Expr Purif. 2002;24(3):348‐356. doi:10.1006/prep.2001.1582 11922750

[25] Sano T , Cantor CR . Streptavidin‐containing chimeric proteins: design and production. Methods Enzymol. 2000;326:305‐311. 11036649 10.1016/s0076-6879(00)26061-8

[26] Gao J , Huang S , Li M , Luo R , Wang X , Takashima A . GM‐CSF‐surface‐modified B16.F10 melanoma cell vaccine. Vaccine. 2006;24(25):5265‐5268. doi:10.1016/j.vaccine.2006.04.031 16713660

[27] Zhang Z , Xu X , Zhang X , et al. The therapeutic potential of SA‐sCD40L in the orthotopic model of superficial bladder cancer. Acta Oncol. 2011;50(7):1111‐1118. doi:10.3109/0284186x.2010.549838 21247263

[28] Nakamura M , Mie M , Funabashi H , Kobatake E . Construction of streptavidin‐luciferase fusion protein for ATP sensing with fixed form. Biotechnol Lett. 2004;26(13):1061‐1066. doi:10.1023/b:bile.0000032966.17759.08 15218380

[29] Hansen M , Met O , Larsen NB , et al. Autocrine CCL19 blocks dendritic cell migration toward weak gradients of CCL21. Cytotherapy. 2016;18(9):1187‐1196. doi:10.1016/j.jcyt.2016.06.010 27424146

[30] Yoshida R , Nagira M , Imai T , et al. EBI1‐ligand chemokine (ELC) attracts a broad spectrum of lymphocytes: activated T cells strongly up‐regulate CCR7 and efficiently migrate toward ELC. Int Immunol. 1998;10(7):901‐910. 9701028 10.1093/intimm/10.7.901

[31] Kellermann SA , Hudak S , Oldham ER , Liu YJ , McEvoy LM . The CC chemokine receptor‐7 ligands 6Ckine and macrophage inflammatory protein‐3 beta are potent chemoattractants for in vitro‐ and in vivo‐derived dendritic cells. J Immunol. 1999;162(7):3859‐3864. 10201903

[32] Cibrian D , Sanchez‐Madrid F . CD69: from activation marker to metabolic gatekeeper. Eur J Immunol. 2017;47(6):946‐953. doi:10.1002/eji.201646837 28475283 PMC6485631

[33] Wieland E , Shipkova M . Lymphocyte surface molecules as immune activation biomarkers. Clin Biochem. 2016;49(4–5):347‐354. doi:10.1016/j.clinbiochem.2015.07.099 26247177

[34] Hombach A , Heuser C , Sircar R , et al. An anti‐CD30 chimeric receptor that mediates CD3‐zeta‐independent T‐cell activation against Hodgkin's lymphoma cells in the presence of soluble CD30. Cancer Res. 1998;58(6):1116‐1119. 9515791

[35] Lanitis E , Poussin M , Hagemann IS , et al. Redirected antitumor activity of primary human lymphocytes transduced with a fully human anti‐mesothelin chimeric receptor. Mol Ther. 2012;20(3):633‐643. doi:10.1038/mt.2011.256 22127019 PMC3293635

[36] Nolan KF , Yun CO , Akamatsu Y , et al. Bypassing immunization: optimized design of "designer T cells" against carcinoembryonic antigen (CEA)‐expressing tumors, and lack of suppression by soluble CEA. Clin Cancer Res. 1999;5(12):3928‐3941. 10632322

[37] Westwood JA , Murray WK , Trivett M , et al. The Lewis‐Y carbohydrate antigen is expressed by many human tumors and can serve as a target for genetically redirected T cells despite the presence of soluble antigen in serum. J Immunother. 2009;32(3):292‐301. doi:10.1097/CJI.0b013e31819b7c8e 19242371

[38] Ma Q , DeMarte L , Wang Y , Stanners CP , Junghans RP . Carcinoembryonic antigen‐immunoglobulin Fc fusion protein (CEA‐Fc) for identification and activation of anti‐CEA immunoglobulin‐T‐cell receptor‐modified T cells, representative of a new class of Ig fusion proteins. Cancer Gene Ther. 2004;11(4):297‐306. doi:10.1038/sj.cgt.7700685 15002034

[39] Schwartzentruber DJ , Hom SS , Dadmarz R , et al. In vitro predictors of therapeutic response in melanoma patients receiving tumor‐infiltrating lymphocytes and interleukin‐2. J Clin Oncol. 1994;12(7):1475‐1483. doi:10.1200/jco.1994.12.7.1475 8021739

[40] Beecham EJ , Ma Q , Ripley R , Junghans RP . Coupling CD28 co‐stimulation to immunoglobulin T‐cell receptor molecules: the dynamics of T‐cell proliferation and death. J Immunother. 2000;23(6):631‐642. 11186151 10.1097/00002371-200011000-00004

[41] Iezzi G , Karjalainen K , Lanzavecchia A . The duration of antigenic stimulation determines the fate of naive and effector T cells. Immunity. 1998;8(1):89‐95. 9462514 10.1016/s1074-7613(00)80461-6

